# A Wandering Liver

**Published:** 1890-12-27

**Authors:** 


### A WANDERING LIVER.

Langenbuch found this almost, if not quite, unique condition in a young woman on whom he had previously operated for wandering kidney. This smaller organ he had fixed by suture, but he endeavoured to secure the liver in its normal position by introducing a large tampon of iodoform mull, which he did not remove for four weeks. The operation appeared to have been successful, but adhesion proved only temporary. Probably suture would have been more effective, and certainly would have been less hazardous, than maintaining an open abdominal wound for a whole month, even "under strict antiseptic precautions."

